# Quantification of patellar tendon reflex using portable mechanomyography and electromyography devices

**DOI:** 10.1038/s41598-021-81874-5

**Published:** 2021-01-27

**Authors:** Hironori Tsuji, Haruo Misawa, Tomoyuki Takigawa, Tomoko Tetsunaga, Kentaro Yamane, Yoshiaki Oda, Toshifumi Ozaki

**Affiliations:** 1 Department of Orthopaedic Surgery, Dentistry, and Pharmaceutical Sciences, Okayama University Graduate School of Medicine, 2-5-1 Shikata-cho, Kitaku, Okayama City, Okayama 700-8558 Japan; 2 Department of Orthopaedic Surgery, Okayama University Hospital, Okayama, Japan; 3 Department of Orthopaedic Surgery, Kobe Red Cross Hospital, Hyogo, Japan

**Keywords:** Neuroscience, Neuronal physiology, Neurological disorders

## Abstract

Deep tendon reflexes are one of the main components of the clinical nervous system examinations. These assessments are inexpensive and quick. However, evaluation can be subjective and qualitative. This study aimed to objectively evaluate hyperreflexia of the patellar tendon reflex using portable mechanomyography (MMG) and electromyography (EMG) devices. This study included 10 preoperative patients (20 legs) who had a pathology that could cause bilateral patellar tendon hyperreflexia and 12 healthy volunteers (24 legs) with no prior history of neurological disorders. We attached MMG/EMG sensors onto the quadriceps and tapped the patellar tendon with maximal and constant force. Our results showed a significantly high amplitude of the root mean square (RMS) and low frequency of the mean power frequency (MPF) in the rectus femoris, vastus medialis, and vastus lateralis muscles in both EMG and MMG with both maximal and constant force. Especially in the patients with cervical and thoracic myelopathy, the receiver operating characteristic (ROC) curve for diagnosing hyperreflexia of the patellar tendon showed a moderate to very high area under the curve for all EMG–RMS, EMG–MPF, MMG–RMS, and MMG–MPF values. The use of EMG and MMG for objectively quantifying the patellar tendon reflex is simple and desirable for future clinical applications and could help diagnose neurological disorders.

## Introduction

Eliciting deep tendon reflexes (DTRs) is one of the main components of the clinical examination of the nervous system. It aids in anatomical diagnosis, which is the essential first step in the neurological diagnostic process, and gives an important indication as to whether a patient’s disorder arises from the central or peripheral nervous system^1^.

Analysis of spinal reflexes is important for the diagnosis of neurological problems^2^. DTRs, such as the patellar tendon reflex (PTR), can be obtained from muscle tendon tapping and show an immediate muscle contraction. The reaction of DTRs differs according to pathologies; hyporeflexia indicates peripheral nervous pathologies, and hyperreflexia indicates central nervous pathologies^3^. Hyperreflexia is a characteristic of several neurological diseases, such as brain stroke, spinal cord injury, and cervical and thoracic myelopathies^4–6^. Therefore, an accurate assessment of the PTR and the objective differentiation of a normal response from an abnormal response are important parts of the clinical assessment. Clinical reflex assessment is inexpensive and quick. However, the evaluation can be subjective and qualitative^7^.

The National Institute of Neurological Disorders and Stroke scale (NINDS) has been proposed as a scoring system to assess tendon reflexes, and joint range of motion was subjectively assessed during the reflex (Table 1)^8^. A previous study suggested that NINDS showed substantial to near-perfect intra-observer reliability^9^. On the other hand, another study showed that the inter-observer reliability was not better than fair, and some disagreement may be related to the experience of the examining physician^10^. However, there are some cases in which the central nervous pathology progresses irreversibly and for which the subjectivity of DTRs would be very stressful for doctors who do not specialize in neurology.

**Table 1:** NINDS for deep tendon reflex assessment.

Score	Description
0	Reflex absent
1+	Reflex slight, less than normal
2+	Reflex in lower half of normal range
3+	Reflex in upper half of normal range
4+	Reflex enhanced, more than normal: includes a clonus

*NINDS* National Institute of Neurological Disorders and Stroke.

To solve this problem, many studies have been conducted using objective methods rather than visual assessment for the quantification of the PTR. Surface electromyography (EMG) has been the most commonly applied method^11,12^ which, while convenient, can be subject to difficulties with respect to electrode placement^13^. Other methods included attaching force transducers^14^, accelerometers^7^, and motion analysis^15,16^. However, these experimental set-ups are difficult to use clinically, and there is little evidence that focuses on the muscle movement itself during the tendon reflex.

A mechanomyography (MMG) is a muscle monitoring technique that measures the mechanical response of the lateral oscillation of a muscle fiber during contraction^17^. Many reports have demonstrated that MMG reflects muscle mechanical activity quantitatively and noninvasively^18–22^. Conventionally, several types of bulky MMG sensors have been developed, such as accelerometers^23^, amorphous sensors^24^, and laser displacement sensors^25^. However, recent technological advances have made available portable-sized MMG photo reflector sensors, thereby making MMG sensors easy to use, both in laboratory and clinical situations^26^.

For the above reasons, a more objective DTR assessment would provide a better diagnosis of neurological problems. We presumed that the MMG device could facilitate the objective assessment of DTRs. This study aimed to objectively evaluate hyperreflexia of the patellar tendon reflex using portable mechanomyography (MMG) and electromyography (EMG) devices.

## Results

### Diagnosis

The hyperreflexia (H) group included nine patients with cervical myelopathy and one with thoracic myelopathy. The H group showed an NINDS score of 3+ in 15 legs and 4+ in 5 legs, whereas the control (C) group showed a score of 2+ in 6 legs and 1+ in 18 legs (Table 2).

**Table 2:** Characteristics of participants.

	H group (n = 10 persons, 20 legs)	C group (n = 12 persons, 24 legs)	*p*-value
Sex	Men, 7; women 3	Men, 9; women, 3	0.73
Age (years)	66.0 ± 8.2	33.2 ± 3.7	0.001
Height (m)	1.59 ± 9.6	1.69 ± 9.26	0.018
Body weight (kg)	60.1 ± 8.2	69.43 ± 14.6	0.101
Body mass index (kg/m^2^)	23.7 ± 2.6	23.8 ± 3.4	0.951
Diagnosis	Cervical myelopathy, 9 Thoracic myelopathy, 1		
**NINDS (legs)**			
1+	0	18	
2+	0	6	
3+	15	0	
4+	5	0	

Data are expressed as number or mean ± standard deviation. The H group included patients with patellar tendon hyperreflexia; the C group included healthy volunteers with normal tendon reflex.

*H* hyperreflexia, *C* control; *NINDS* The National Institute of Neurological Disorders and Stroke.

### Experiment 1: PTR movement with maximal tap force

**Figure 1:**
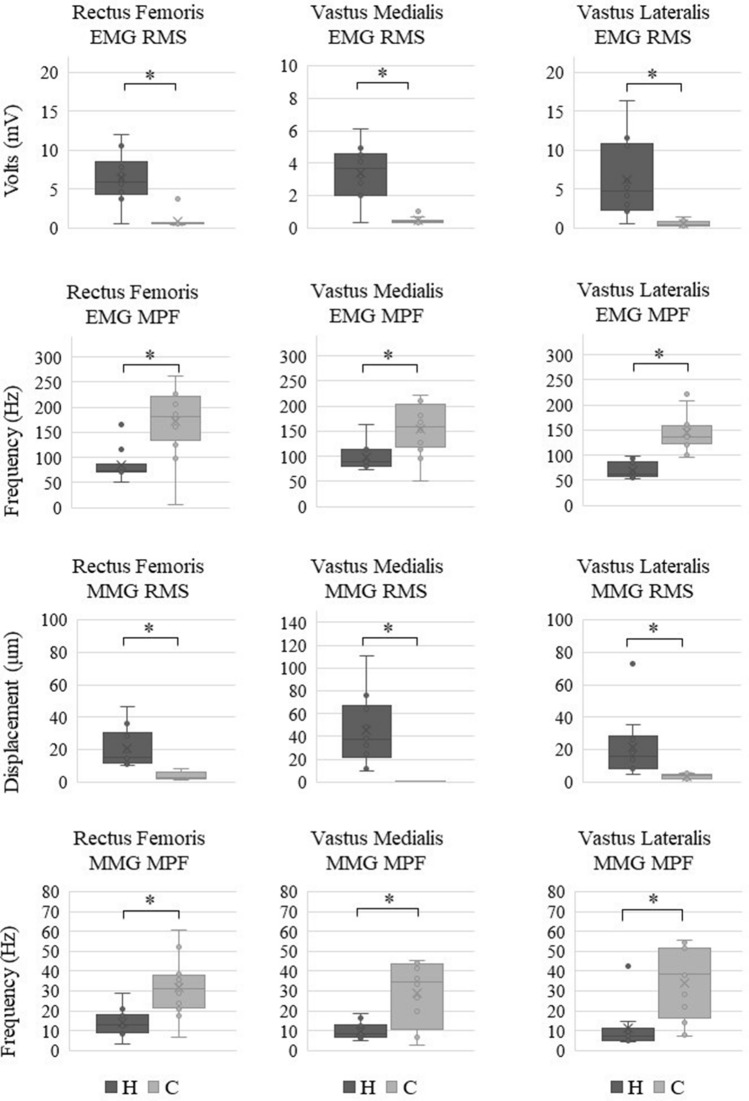
Differences between participants with hyperreflexia (H group) and control participants (C group) for EMG–RMS, EMG–MPF, MMG–RMS, and MMG–MPF with maximal force. The H group is shown on the left side, and the C group is shown on the right side of each graph. Standard deviation is shown as an error bar. The asterisk indicates *p* < 0.001. EMG: electromyography, RMS: root mean square, MMG: mechanomyography, MPF: mean power frequency.

The H group showed significantly higher amplitudes in the root mean square (RMS) and lower frequencies in the mean power frequency (MPF) in the rectus femoris (RF), vastus medialis (VM), and vastus lateralis (VL) for both EMG and MMG than the C group (Fig. 1).

### Experiment 2: PTR movement with constant tap force

**Figure 2:**
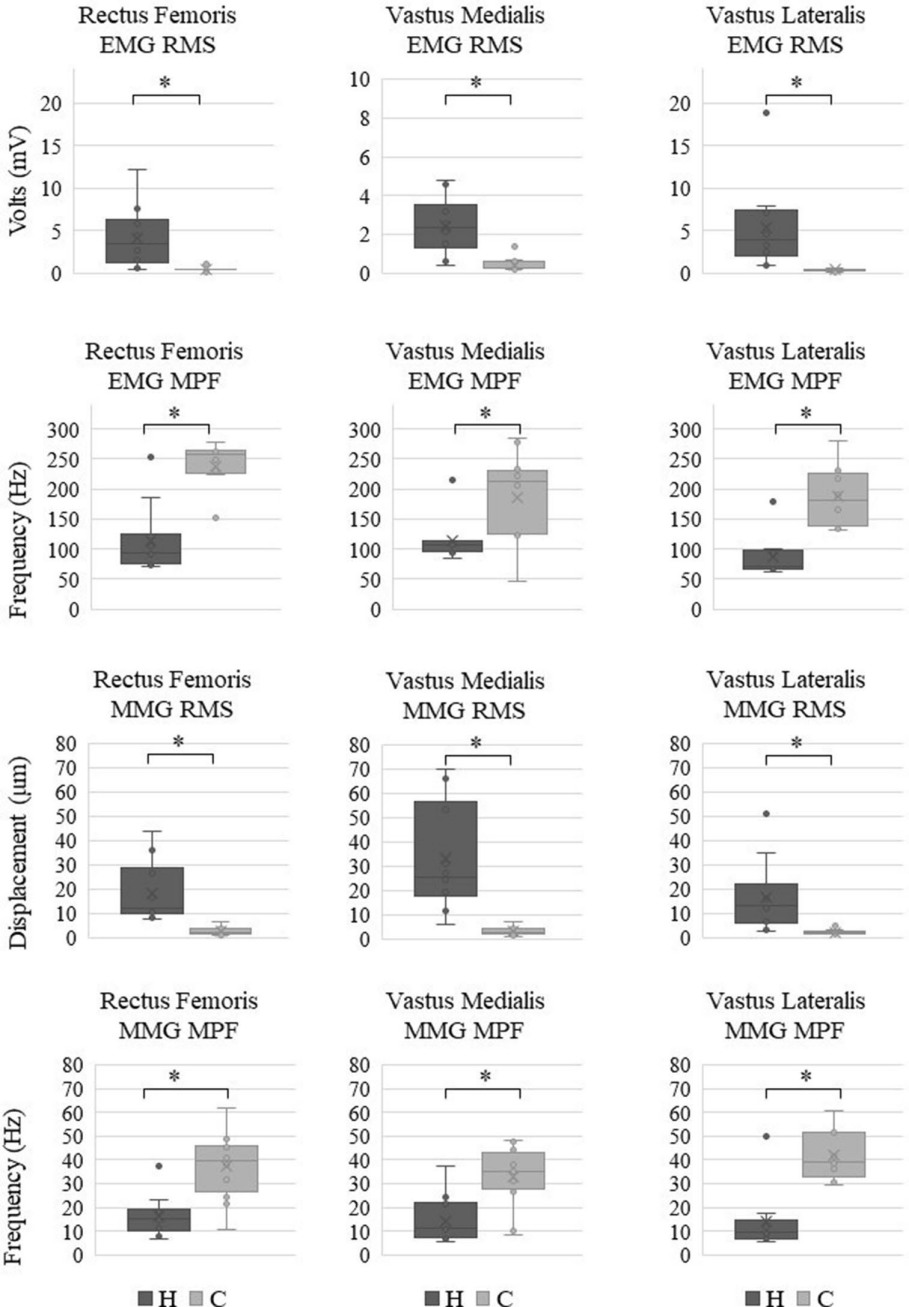
Differences between participants with hyperreflexia (H group) and control participants (C group) for EMG–RMS, EMG–MPF, MMG–RMS, and MMG–MPF with a constant force. The H group is shown on the left side, and the C group is shown on the right side of each graph. Standard deviation is shown as an error bar. The asterisk indicates *p* < 0.001. EMG: electromyography, RMS: root mean square, MMG: mechanomyography, MPF: mean power frequency.

The mean tap force was 142.6 ± 7.0 N using our tool. When tapped by our tool, the H group showed significantly higher amplitudes in RMS and lower frequencies in MPF in all muscles for both EMG and MMG than the C group (Fig. 2).

### ROC curve

The ROC curve and area under the curve (AUC) of EMG–RMS, EMG–MPF, MMG–RMS, and MMG–MPF were obtained for each muscle. In experiment 1, the AUC of each parameter was over 0.9 in the RF and VL. In the VM, the AUC of EMG–RMS and MMG–RMS was over 0.9 and that of EMG–MPF and MMG–MPF was 0.82 and 0.79, respectively. In experiment 2, the AUC of each parameter was over 0.9 in the three muscles. The cut-off respective values in the RF, VM, and VL muscles were as follows: MMG–RMS: 7.80, 10.00, and 6.10 and MMG–MPF: 23.30, 24.20, and 15.20 in experiment 1, and MMG–RMS: 7.53, 10.23, and 3.37 and MMG–MPF; 27.67, 27.67, and 20.03 in experiment 2 (Table 3).

**Table 3:** Diagnostic parameters of the ROC curve for each parameter.

	Cut-off value	Specificity	Sensitivity	AUC (95%CI)
**Experiment 1 maximal force**				
RF				
EMG				
RMS	1.50	0.96	0.95	0.97 (0.93–1.00)
MPF	129.7	0.83	0.95	0.94 (0.85–1.00)
MMG				
RMS	7.8	0.92	1.00	0.99(0.98–1.00)
MPF	23.30	0.79	0.95	0.92(0.83–1.00)
VM				
EMG				
RMS	1.10	1.00	0.95	0.96 (0.89–1.00)
MPF	117.8	0.71	0.95	0.82 (0.68–0.96)
MMG				
RMS	10.00	0.88	1.00	0.98 (0.95–1.00)
MPF	24.20	0.67	1.00	0.79 (0.64–0.94)
VL				
EMG				
RMS	2.00	1.00	0.90	0.98 (0.94–1.00)
MPF	99.50	0.88	1.00	0.98 (0.94–1.00)
MMG				
RMS	6.10	0.96	0.95	0.98 (0.96–1.00)
MPF	15.20	0.88	0.95	0.92 (0.83–1.00)
**Experiment 2 constant force**				
RF				
EMG				
RMS	1.07	1.00	0.85	0.96 (0.90–1.00)
MPF	124.1	0.96	0.85	0.93 (0.85–1.00)
MMG				
RMS	7.53	0.96	0.95	0.99 (0.97–1.00)
MPF	27.67	0.83	0.95	0.93 (0.85–1.00)
VM				
EMG				
RMS	0.60	0.88	0.95	0.97 (0.93–1.00)
MPF	114.33	0.88	0.95	0.90 (0.80–1.00)
MMG				
RMS	10.23	1.00	0.95	1.00 (0.99–1.00)
MPF	27.67	0.83	0.95	0.92 (0.84–1.00)
VL				
EMG				
RMS	0.87	1.00	0.95	0.98 (0.94–1.00)
MPF	113.87	0.96	0.90	0.95 (0.89–1.00)
MMG				
RMS	3.37	0.92	0.90	0.96 (0.92–1.00)
MPF	20.03	0.92	0.95	0.95 (0.87–1.00)

Data of cut-off value are shown as mV for EMGRMS, µm for MMGRMS, and Hz for EMGMPF and MMGMPF.

*ROC* receiver operating characteristic, *AUC* area under the curve, *CI* confidence interval, *RF* rectus femoris, *VM* vastus medialis, *VL* vastus lateralis, *EMG* electromyography, *MMG* mechanomyography, *RMS* root mean square, *MPF* mean power frequency.

### Power analysis

The power analysis of EMG–RMS, EMG–MPF, MMG–RMS, and MMG–MPF was obtained for each muscle. For both experiments 1 and 2, the effect sizes and power of each parameter were over 0.8, indicating that the effect size was large and the sample sizes of the two experiments were sufficient (Table 4).

**Table 4:** Effect size, power, and p-values for each parameter.

	Effect size (95% CI)	Power	*p*-value
**Experiment 1 maximal force**			
RF			
EMG			
RMS	1.22 (0.91 – 1.53)	0.98	0.000
MPF	1.20 (0.89 – 1.51)	0.97	0.000
MMG			
RMS	1.19 (0.88 – 1.50)	0.97	0.000
MPF	1.17 (0.96 – 1.48)	0.97	0.000
VM			
EMG			
RMS	1.20 (0.89 – 1.51)	1.06	0.000
MPF	1.06 (0.75 – 1.37)	0.97	0.000
MMG			
RMS	1.13 (0.82 – 1.44)	0.95	0.000
MPF	1.10 (0.79 – 1.41)	0.94	0.000
VL			
EMG			
RMS	1.15 (0.79 – 1.41)	0.96	0.000
MPF	1.22 (0.91 – 1.53)	0.98	0.000
MMG			
RMS	1.11 (0.80–1.42)	0.95	0.000
MPF	1.17 (0.86–1.48)	0.97	0.000
**Experiment 2 constant force**			
RF			
EMG			
RMS	1.13 (0.82–1.44)	0.95	0.000
MPF	1.21 (0.90 – 1.52)	0.97	0.000
MMG			
RMS	1.17 (0.86–1.48)	0.97	0.000
MPF	1.19 (0.88–1.50)	0.97	0.000
VM			
EMG			
RMS	1.17 (0.86–1.48)	0.97	0.000
MPF	1.12 (0.81–1.43)	0.95	0.000
MMG			
RMS	1.15 (0.84–1.46)	0.96	0.000
MPF	1.21 (0.90–1.52)	0.97	0.000
VL			
EMG			
RMS	1.13 (0.82–1.44)	0.95	0.000
MPF	1.20 (0.89 – 1.51)	0.97	0.000
MMG			
RMS	1.12 (0.81–1.43)	0.95	0.000
MPF	1.22 (0.91–1.53)	0.98	0.000

*CI* confidence interval, *RF* rectus femoris, *VM* vastus medialis, *VL* vastus lateralis, *EMG* electromyography, *MMG* mechanomyography, *RMS* root mean square, *MPF* mean power frequency.

### Intraclass correlation (ICC) and standard error of measurement (SEM)

The ICCs of H and C group of EMG–RMS, EMG–MPF, MMG–RMS, and MMG–MPF for maximal tendon reflex were obtained for each muscle (Table 5). H group showed moderate reliability in EMG–MPF of the VL, and almost perfect reliability for all other parameters. C group showed substantial reliability for EMG–RMS and MMG–MPF of the RF, EMG–RMS of the VL, and almost perfect reliability for all other parameters. The SEMs of each parameter was calculated and shown on Table 5.

**Table 5:** ICC and SEM of maximal tendon reflex for each parameter.

	ICC	ICC (95% CI)	SEM
**H group**			
RF			
EMG			
RMS	0.97	0.92–0.99	0.53
MPF	0.89	0.75–0.96	14.33
MMG			
RMS	0.96	0.91–0.98	2.42
MPF	0.84	0.64–0.93	2.65
VM			
EMG			
RMS	0.95	0.87–0.98	0.45
MPF	0.84	0.64–0.93	10.97
MMG			
RMS	0.92	0.82–0.97	6.75
MPF	0.93	0.84–0.97	1.92
VL			
EMG			
RMS	0.97	0.93–0.99	0.73
MPF	0.56	0.18–0.80	16.35
MMG			
RMS	0.95	0.89–0.98	3.22
MPF	0.89	0.75–0.96	3.17
**C group**			
RF			
EMG			
RMS	0.78	0.56–0.90	0.14
MPF	0.92	0.84–0.97	10.82
MMG			
RMS	0.91	0.80–0.96	0.36
MPF	0.79	0.58–0.90	4.37
VM			
EMG			
RMS	0.88	0.75–0.96	0.04
MPF	0.95	0.88–0.98	9.41
MMG			
RMS	0.94	0.86–0.97	0.58
MPF	0.85	0.68–0.93	4.05
VL			
EMG			
RMS	0.70	0.427–0.86	0.11
MPF	0.83	0.65–0.92	11.26
MMG			
RMS	0.84	0.66–0.93	0.35
MPF	0.86	0.71–0.94	4.11

Data of SEM are shown as mV for EMGRMS, µm for MMGRMS, and Hz for EMGMPF and MMGMPF.

*ICC* interclass correlation, *SEM* standard error of measurement, *CI* confidence interval, *RF* rectus femoris, *VM* vastus medialis, *VL* vastus lateralis, *EMG* electromyography, *MMG* mechanomyography, *RMS* root mean square, *MPF* mean power frequency.

## Discussion

The present study has shown that in patients with PTR hyperreflexia, both EMG and MMG have a higher RMS and a lower MPF than those in normal participants with respect to all three quadriceps muscles—RF, VM, and VL. Especially in the patients with cervical and thoracic myelopathy, the ROC curve for diagnosing hyperreflexia assessed by MINDS of the patellar tendon showed a moderate to very high AUC for all EMG–RMS, EMG–MPF, MMG–RMS, and MMG–MPF values.

A deep tendon reflex is the contraction of a muscle resulting from tapping or stretching the muscle spindles. The tendon reflex is composed of a two-neuron arc. The afferent neuron is innervated when the muscle spindle is tapped or stretched and excites alpha motor neurons in the anterior horn of the spinal cord. The tapped muscle is contracted via this alpha motor neuron^27^.

In the PTR, a tapping force to the patellar tendon stretches the spindles of the quadriceps, enters the spinal cord from the dorsal root of the spinal cord by the sensory nerves, reaches the anterior root through the reflex arc, and contracts the quadriceps muscles via the motor nerves, resulting in knee extension.

Clinically, the examinee was determined to be hyperactive or normal depending on the strength of the examiner’s impact on the patellar tendon with a hammer, and both the extent of knee extension and tap force were subjective. Many objective methods of PTR evaluation and quantification have been reported. EMG latency and EMG amplitude during PTR have been previously reported^11,28^. As it was difficult to evaluate the EMG latency in this study, the amplitude was used. A previous study showed that the EMG amplitude and torque were stronger in spinal cord injury patients than in healthy volunteers^29^, consistent with our EMG results. Other devices have also been used to evaluate the PTR response. An accelerometer was used to demonstrate the difference between myelopathy patients and healthy volunteers in terms of the PTR response^7^, and another study reported an accuracy of 89.62%, according to the NINDS score, using machine learning classifiers^30^. A motion capture system was also used to demonstrate the knee angle increase dependent on the tapping force in healthy participants^15,16^ and its usability for cerebral palsy patients^31^. As for MMG, there was no evidence of PTR quantification. The merits of our study over previous reports were related to portability and objectivity. The MMG device used in this study was portable and wireless, which means the possibility of clinical usage at the bedside, even for patients with gait disturbance. Regarding objectivity, this method has merit in that it places the MMG directly above the quadriceps muscles, so that the movements of the quadriceps muscles could be captured directly without being affected by individual differences in the weight of the lower limbs or range of motion of the knee. As for the assessment of muscle movement, this method would be more objective than visual assessment and other methods. To tap more objectively, we used a tool that could hit the patellar tendon with a constant force through a method of dropping a fixed weight from a certain angle using gravity. As has been previously reported, the maximal force that provokes maximal PTR movement differs individually, and there were S-shaped relationships between the stimulus power and response. In other words, under maximal force, a larger force generated a larger response, and the response became constant above the maximal force^12,13^. We intended to analyze maximal movement over maximal force using a hammer and submaximal force with constant force. Then, the largest waveform for maximal force and the average of three waveforms for constant force were selected and analyzed. The reason for the difference in wave analysis was as follows: For maximal force, a slight change in the tapping point could change the PTR response. In addition, examiners may unconsciously hesitate to apply repeated maximal force to patients. Therefore, we used the largest waveforms for maximal force. For constant force, we placed a custom-made device on the skin, so that the tapping point was more stable than maximal force. Then, we used the average of the three waveforms for constant force.

The merits of measuring both the EMG and MMG were that they could accurately evaluate the quadriceps muscle contraction after patellar tendon percussion and could prevent the evaluation of surface movement unrelated to the muscle movement. Tapping of the patellar tendon stimulated the EMG from the sensory and motor neurons, and MMG was stimulated by the quadriceps muscle contraction evoked by the activated motor neuron. Subsequently, if the EMG waveforms were not detected, it was thought to be noise from the surface movement. Moreover, to avoid detecting active muscle movement, we checked the active knee extension and waveforms during set-ups. The waveforms of the tendon reflex were sharp and narrow, whereas those of active muscle contraction were dull and wide. We assessed only the sharp wave, which was thought to be a result of PTR movement during data processing.

With respect to waveform analysis, previous studies have used both EMG and MMG to evaluate muscle contraction^26,32,33^. In these methods, the MMGRMS and MMGMPF are generally used for analysis, and we also used a similar method. To perform objective waveform analysis, both EMG and MMG peaks were detected automatically, and the range of 0.5 s from the peak was analyzed. For peak detection, we also checked the waveform on the computer screen to determine if an appropriate waveform could be detected. The timing of peak EMG and MMG of each muscle contraction during PTR might not be shown simultaneously and was not evaluated in this study. However, both peaks of MMG and EMG were thought to represent the muscle response of PTR, and we analyzed each peak waveform. As the contraction of the quadriceps femoris was greater in the participants with hyperreflexia than in healthy individuals, waveforms of both EMG and MMG during PTR were large and resulted in a larger RMS. As for MPF, the lower frequency component was larger in the participants with hyperreflexia than in healthy individuals. A previous study revealed that the MMG frequency component of slow muscle was smaller than that of the fast muscle^34^. Our study analysed the MMG waveform after peak muscle movement, which was thought to consist of slow muscle movement. We considered the results about frequency analysis as follows: the electric signal evoked by patellar tendon tapping in group H was greater; more slow muscles, which consist of a lower-frequency component, might have been evoked and resulted in lower MPF in group H than in group C. However, the relationships between frequency domain analyses and muscle contraction need further study, deeming this a limitation of our study.

As the sensors used in this study were small and light, the muscles could be selectively evaluated. We evaluated each component of the quadriceps muscle by placing sensors on the surface of each muscle, and the results were the same for all muscles. This indicates that for the cervical and thoracic myelopathy participants, three muscles of 20 legs had the same neurological aspects during PTR. Therefore, when considering the clinical use, one of the muscles might be representative.

Although this study comprised a sufficient number of cases by power analysis, the patients’ diseases were limited to spinal pathologies. Thus, it is necessary to include more cases of spinal as well as other pathologies. Moreover, there are some healthy people whose tendon reflexes are enhanced without any neurological past history, especially in young people^35^. Conversely, even in patients suspected to have upper motor neuron disorders, PTR is not enhanced when peripheral neuropathic diseases, such as diabetes mellitus^36^, coexist. These atypical cases have not been examined in this study. Therefore, it might be difficult to distinguish pathological hyperreflexia from healthy hyperreflexia of the PTR and vice versa, which is one of the limitations of our study.

The Jendrassik maneuver is a method used for increasing the tendon reflexes. However, we did not use this method in this study for two reasons. First, patients with hyperreflexia showed increased patellar tendon reflex, and the effect of the Jendrassik maneuver in patients with hyperreflexia is uncertain. Second, the effect of the Jendrassik maneuver reportedly decreases with age^35^, and in this study, we included older participants with hyperreflexia and younger healthy volunteers. This was another limitation of this study.

A third limitation of this study also pertained to the age of the participants. We included older participants with central nervous pathology and younger healthy volunteer because it was difficult to establish a healthy, older cohort and vice versa. A previous study showed that aging significantly decreases the reflex response^37^. In this study, as older patients showed a higher reflex response than younger volunteers, the decreased effect of aging on the reflex response might not change the result of this study. Nevertheless, it was a major limitation of this study, and it is clear that further evidence should be accumulated, including the characteristics of age, height, and sex.

As another limitation, quadriceps muscle volume and subcutaneous thigh fat may affect MMG results. A previous study reported that subcutaneous fats attenuate MMG signals and act as a filter, which dissipates high-frequency components^38^. In our study, as body mass index of the H and C group had not significantly different, we did not perform local assessment of thigh fat and muscle which would affect our results. It was limitation of this study.

Despite the above limitations, the method used in this study could enable objective PTR evaluation as it is a simple method that can be set up in a short time, which may be useful even in busy clinical situations. In conclusion, the EMG and MMG waveforms of the PTR were significantly different between the hyperreflexia of participants with pathologies and that of normal participants. The use of EMG and MMG for objectively quantifying the PTR is simple and desirable for future clinical applications, which could help the diagnosis of neurological disorders. In the future, a larger number of patients with spinal pathologies and other neurological pathologies will be needed to expand these results.

## Methods

### Participants

The present study was performed at the first author’s institution and conformed with the principles outlined in the Declaration of Helsinki of 1964. Ethical approval was obtained from the Ethics Committee of Okayama University Graduate School of Medicine, Dentistry and Pharmaceutical Sciences and Okayama University Hospital (No. 1911-001). Participants included 10 preoperative patients (20 legs, age: 62.0 ± 8.23 years, seven men, three women) who had pathologies that could cause bilateral patellar tendon hyperreflexia and 12 healthy volunteers (24 legs, age: 33.83 ± 4.71 years, nine men, three women) with no prior history of neurological disorders (Table 2). The PTR was evaluated using the NINDS scale (Table 1). Participants with an NINDS tendon reflex score of 3+ and 4+ were classified under the hyperreflexia (H) group, and those with a normal tendon reflex score (1+) and (2+) were classified under the control (C) group. The assessment was performed consecutively for each leg of each participant on the same day. The diagnosis of each patient was made using imaging, including magnetic resonance imaging and computed tomography, and the physical examination was performed by a spine specialist approved by the Japanese Society for Spine Surgery and Related Research. The imaging analysis and physical examination were performed as a preoperative evaluation on a different day. Informed consent was obtained from each participant.

### Experimental procedures

**Figure 3:**
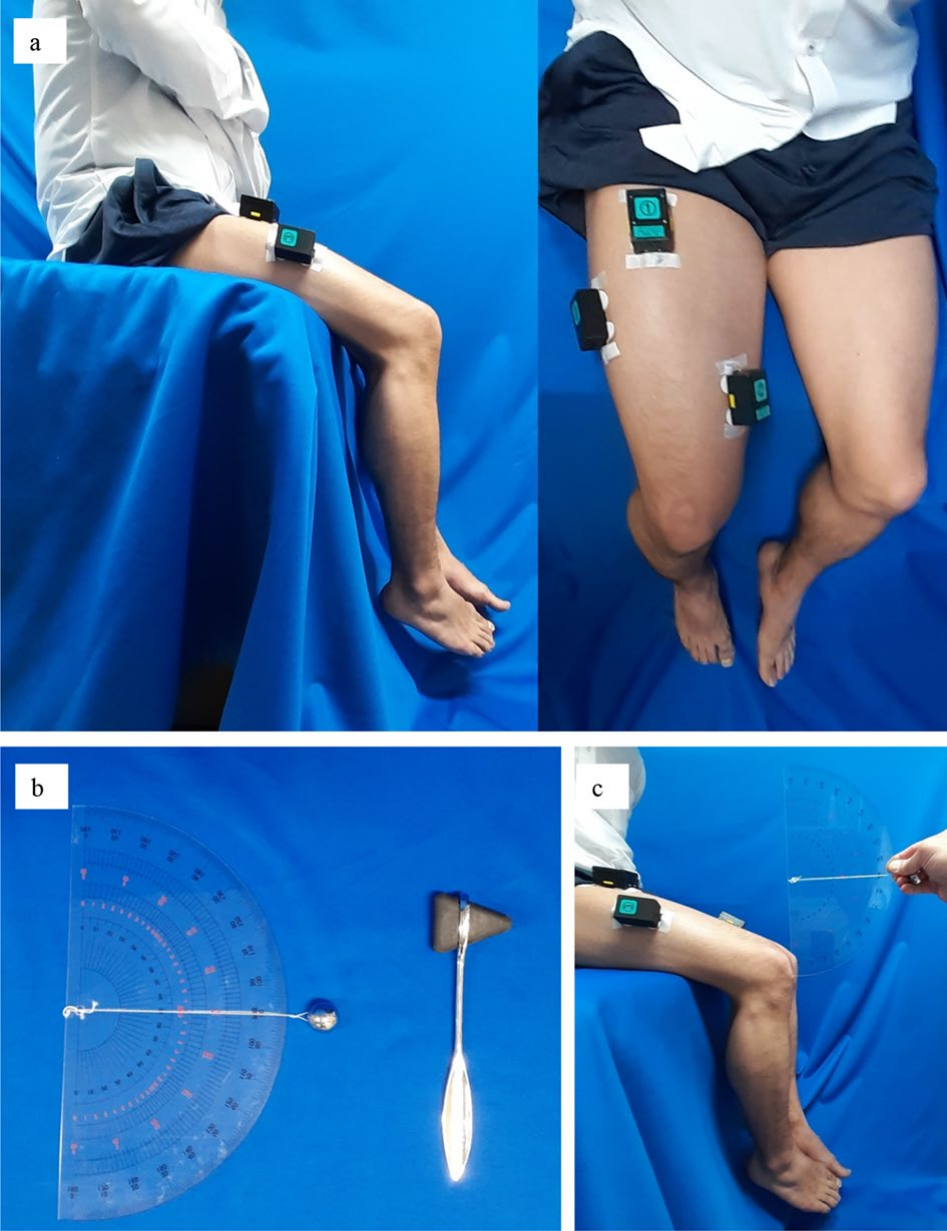
(**a**) Sensors and attachments. Three sensors were placed on the quadriceps, rectus femoris, vastus medialis, and vastus lateralis muscles. (**b**) Hammer (right) and the tool (left) used to evoke the patellar tendon reflex. (**c**) An iron ball was attached using string to a goniometer and the iron ball was dropped from 90° onto the patellar tendon using gravity.

**Figure 4:**
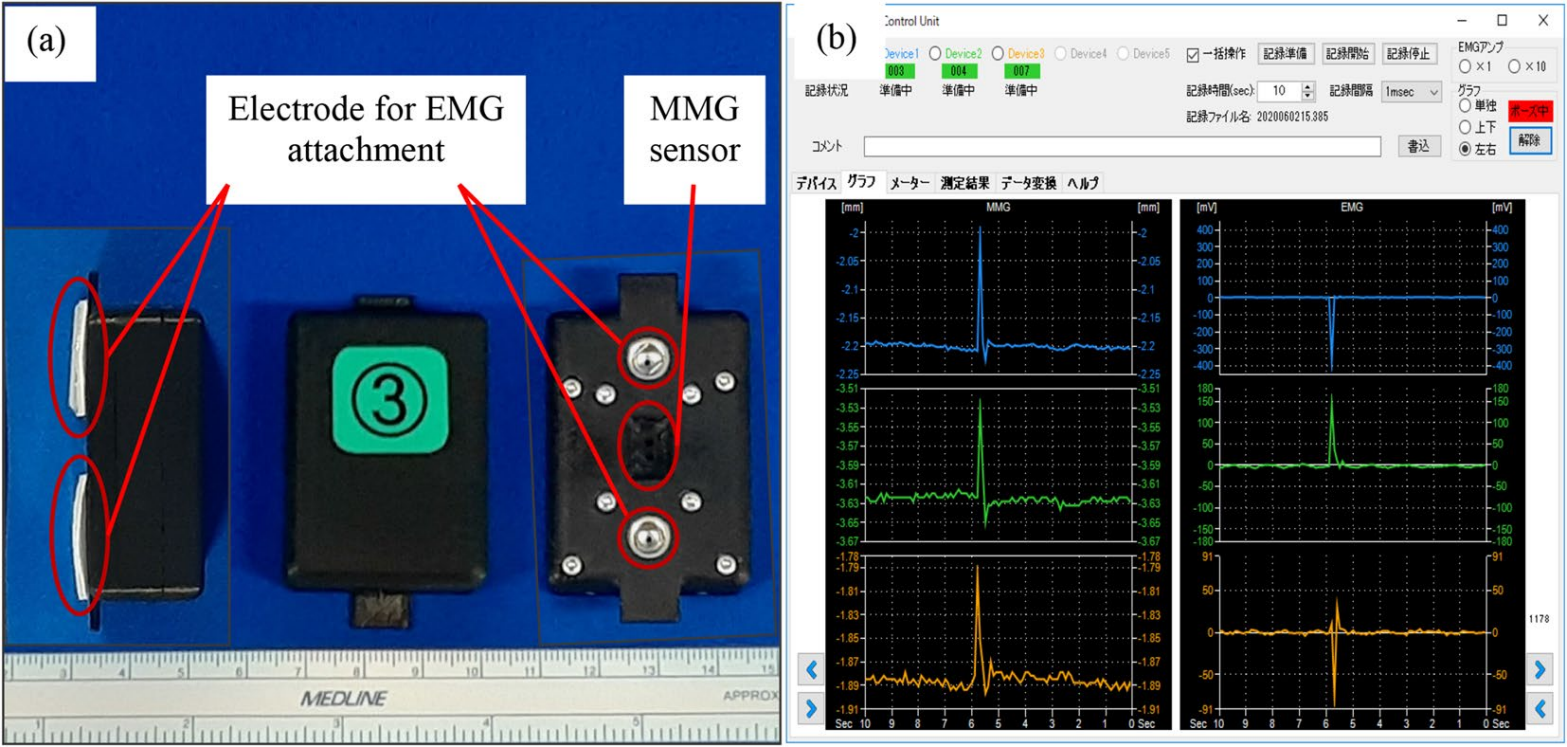
(**a**) Sensor size was 47 × 34 × 24 mm. A photo reflector-based MMG sensor was located in the center of the device. Disposable electrodes were attached to the bottom of the transducer. (**b**) Measurement and control software for operating the MMG/EMG sensor. The waveforms of MMG (left) and EMG (right) are shown on the screen. Up to five sensors could be controlled simultaneously. EMG: electromyography, MMG: mechanomyography.

With the subject sitting upright on a seat with a hip angle of 80° and a knee angle of 70°, three sensors were placed on the quadriceps: RF at 50% distance on the line from the anterior spina iliaca superior to the superior part of the patella, VM at 80% distance on the line between the anterior spina iliaca superior and the joint space in front of the anterior border of the medial ligament, and VL at 66% distance on the line from the anterior spina iliaca superior to the lateral side of the patella, as recommended by the Surface ElectroMyoGraphy for the Non-Invasive Assessment of Muscles project. The EMG/MMG electrode sites were shaved and abraded before applying the adhesive. The EMG/MMG devices were secured to the thigh with tape (Fig. 3a). The EMG/MMG system used was the Measee system (NCP Inc., Okayama, Japan) (Fig. 4a). The transducer measured 47 × 34 × 24 mm and weighed 34 g. The MMG sensor used a photo reflector (TCRT1000, Vishay Intertechnology, Inc., USA). The photo reflector was designed to be 3 mm above the skin surface, and skin variations according to changes in the cross-sectional area were recorded as the MMG. EMG was measured by attaching disposable electrodes to the bottom of the transducer. The measurement and control software program was installed on a personal computer and used to control the transducer, and the transducer and computer terminal communicated via Bluetooth (ver. 4.0) (Fig. 4b). The waveforms of both MMG and EMG were shown in real-time and recorded on an SD card inserted in the sensor. The set measurement time and sampling frequency were 20–30 s and 1,000 Hz, respectively, for each experiment. Data stored on the SD card were converted to μm (MMG) and mV (EMG) using the software and saved on a personal computer, whose measurement error was ± 2.9 μm for MMG and ± 0.427 μV for EMG. Before each experiment, active knee motion was ordered, and waveforms were confirmed visually on a computer. After confirmation of the set-ups, two experiments were performed for each participant.

*Experiment 1* To confirm the waveforms during maximal PTR movement, we employed a hammer utilized in clinical situations. Immediately before the experiment, the hitting force that evoked the maximal PTR was confirmed, and the waveforms evoked when the patellar tendon was hit with that same force were recorded.

*Experiment 2* To hit the patellar tendon with a constant force, we constructed a tool consisting of an iron ball (diameter, 2 cm; weight, 45 g) attached via a string to a goniometer (diameter, 30 cm) (Fig. 3b). The tapping force of the tool was evaluated using Force Gauge (ZTA-500N, Imada Inc, Japan). One end of the goniometer was fixed 2 cm proximal to the patellar tendon, and the iron ball was dropped at an angle of 90° onto the patellar tendon (Fig. 3c). The waveforms of the PTR following each hit by this tool were recorded. All percussions for each experiment were performed more than three times, and both legs of each participant were evaluated. Care was taken to minimize artifact during the PTR evaluation.

### Data processing

**Figure 5:**
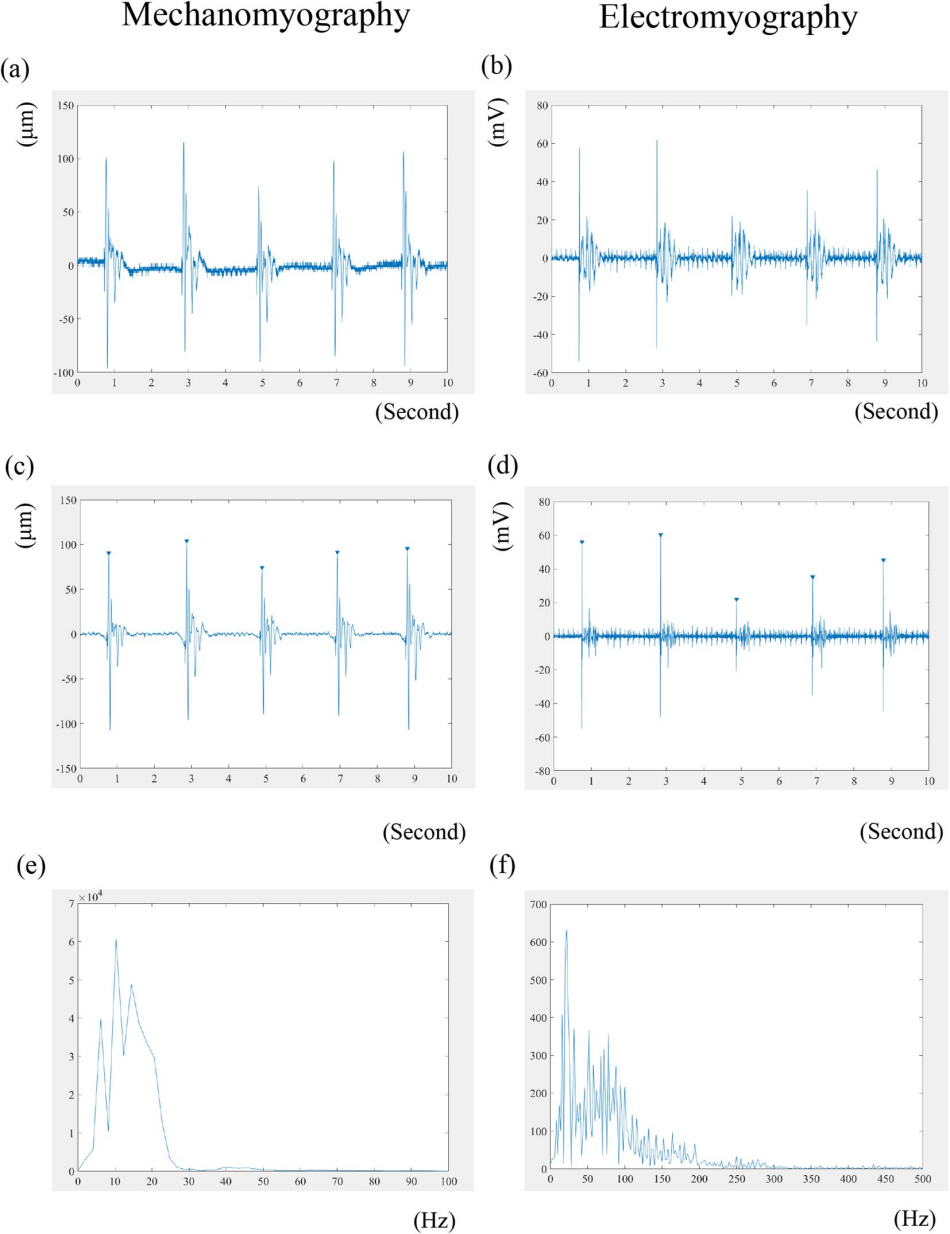
Wave analyses. Typical raw waveform for MMG (**a**) and EMG (**b**). Filtering and peak detection for MMG (**c**) and EMG (**d**). An inverted triangle is shown on each peak waveform, and a point 0.5 ms after each peak was selected; subsequently, RMS was evaluated. MPF was evaluated using a fast Fourier transformation algorithm for MMG (**e**) and EMG (**f**). MMG, mechanomyography; EMG, electromyography; RMS, root mean square; MPF, mean power frequency.

**Figure 6:**
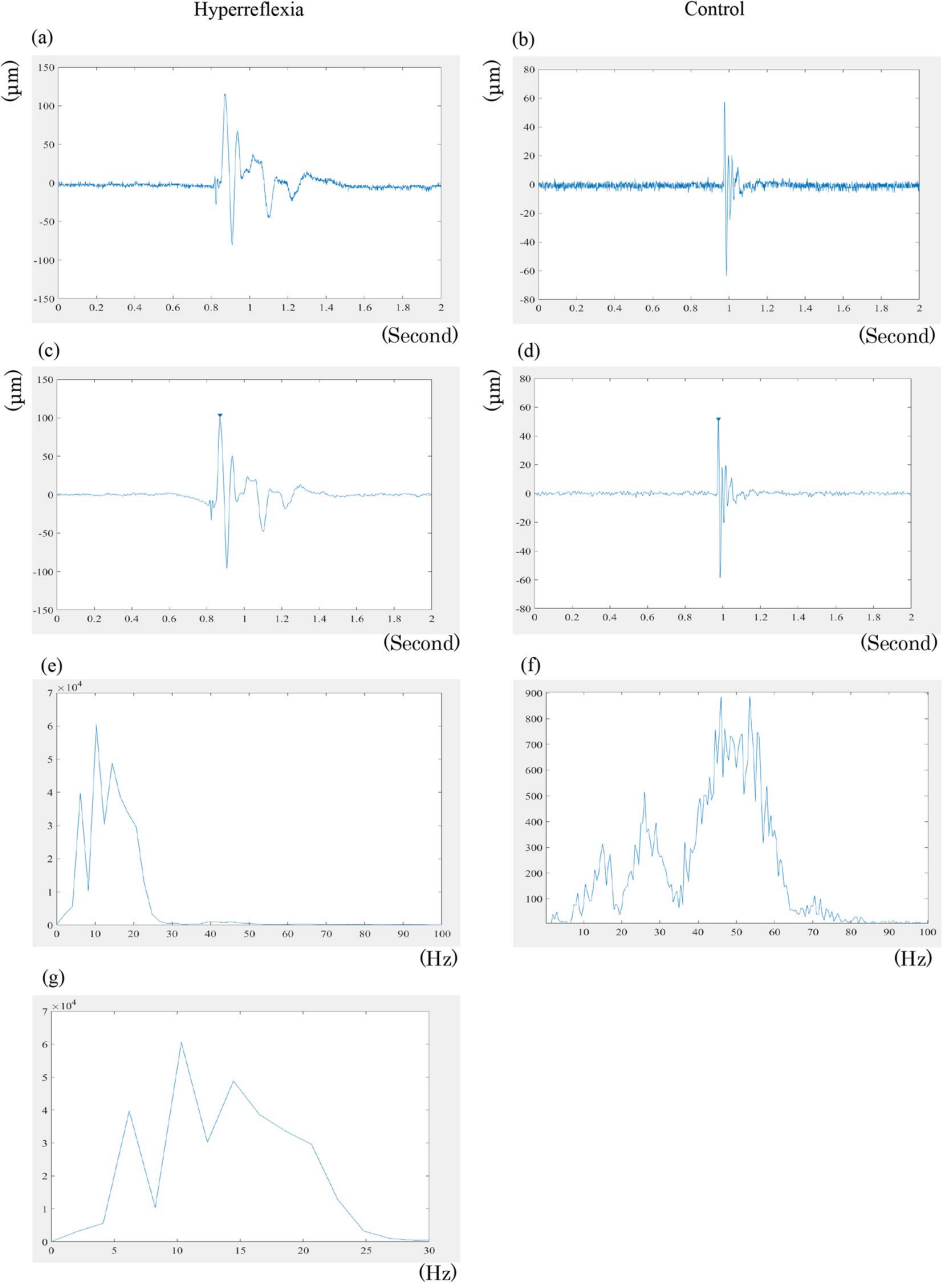
Typical MMG waveform. The patellar tendon reflex's typical raw waveform with hyperreflexia (**a**) and control (**b**). After 2–100 Hz bandpass filtering of hyperreflexia (**c**) and control (**d**). The inverted triangle is shown on the peak waveform; a point 0.5 ms after the peak wave was selected for analysis, and the RMS of this period was evaluated. A fast Fourier transformation algorithm was used for frequency analysis, and the frequency component is shown for hyperreflexia (**e**) and control (**f**). (**g**) The component below 30 Hz shows the lower frequency component for hyperreflexia. MMG, mechanomyography; RMS, root mean square.

**Figure 7:**
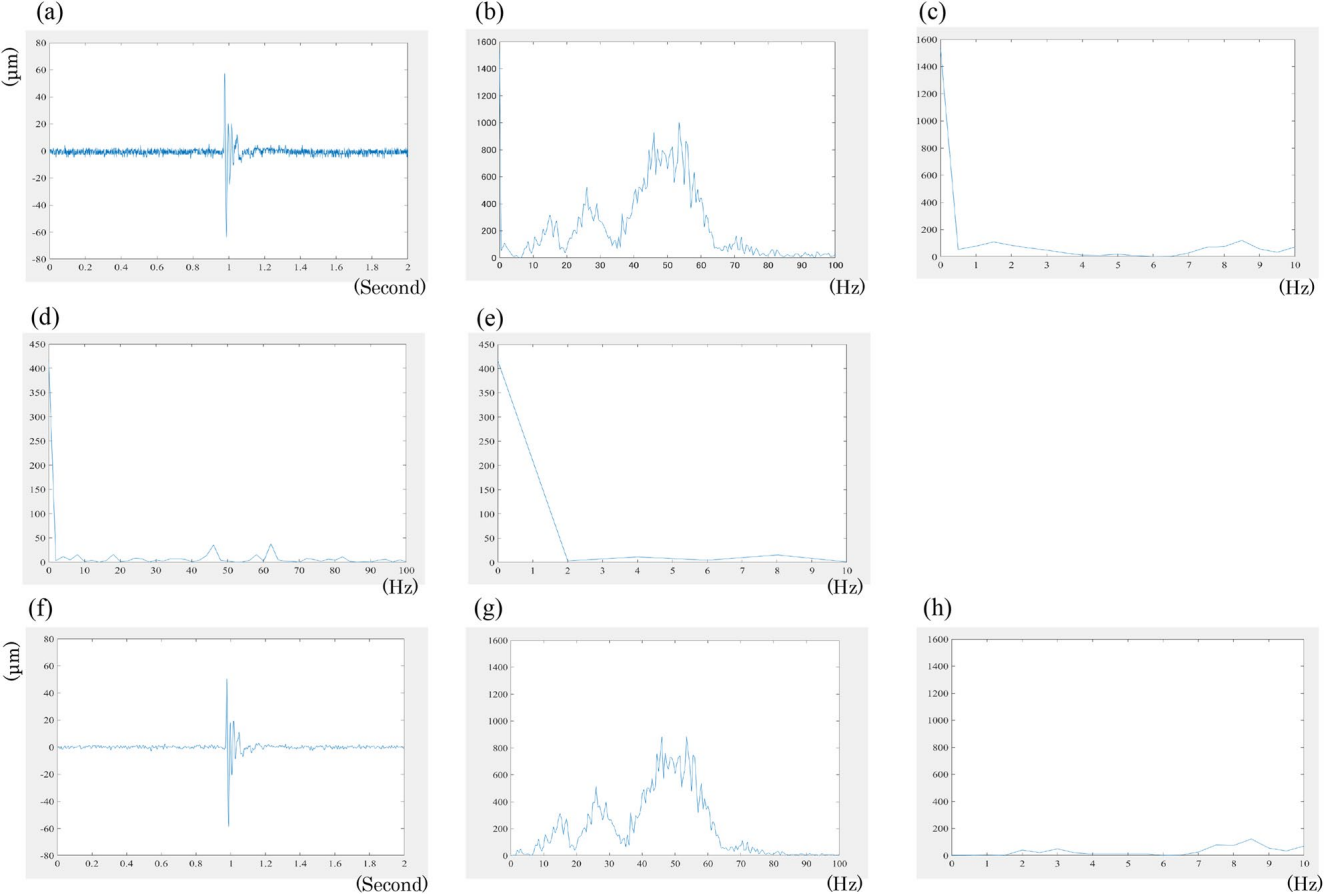
(**a**) A typical raw MMG waveform. Frequency analysis using an FFT algorithm for the raw waveform of the 0.5 ms after the peak wave (**b**) and below 10 Hz (**c**). FFT for the baseline waveform (**d**) and below 10 Hz (**e**); noise was included below 2 Hz. (**f**) An MMG waveform after filtering through a 2–100 Hz bandpass filter. Next, FFT for the waveform of the 0.5 ms after the peak was performed for frequency analysis (**g**) and below 10 Hz (**h**); noise was excluded. MMG, mechanomyography; FFT, fast Fourier transformation.

During offline processing, the raw data were processed as shown in Fig. 5 (EMG and MMG) and Fig. 6 (MMG). The raw MMG data were band-pass filtered between 2 and 100 Hz, whereas the raw EMG data were band-pass filtered between 10 and 500 Hz using a fourth-order Butterworth filter as was performed in previous studies^33,39^. Added on this previous study, as the area under 2 Hz included much noise, lower threshold of 2 Hz for filtering was selected (Fig. 7). With respect to both the MMG and EMG, the peak waves were detected automatically and checked visually. To exclude the input signal when tapping the tendon in the waveform for both the EMG and MMG, the peak waveform at the point of maximal activated motor neuron for EMG and at the point of maximal quadriceps femoris contraction for MMG was taken as the starting point, and the duration from that point was analyzed. We analyzed the waveforms at 0.5 ms after each peak, and the RMS and MPF values of this period were calculated. RMS values were calculated using following equation:

1 $$ {\text{RMS}} = \sqrt {\frac{1}{{T_{2} - T_{1} }}\int_{{T_{1} }}^{{T_{2} }} {\left[ {x\left( t \right)} \right]^{2} } dt} $$

where ${T}_{1}$ is time of peak waveform, ${T}_{2}$ is time of 0.5 ms after peak, $x\left(t\right)$ is the amplitude (mV) for EMG and displacement (μm) for MMG signal.

For the frequency domain analysis, a fast Fourier transformation algorithm was used to quantify the resulting power spectrum of the EMG and MMG signals and MPF was calculated using following equation:

2 $$ {\text{MPF}} = { }\frac{{\mathop \sum \nolimits_{i = 0}^{H} f_{i} P_{i} }}{{\mathop \sum \nolimits_{i = 0}^{H} P_{i} }} $$

where $H$ is the highest cut-off frequency, $Pi$ is the ith line of the power spectrum, and $fi$ is the frequency variable. The largest wave for experiment 1 and the average of three waves for experiment 2 were selected and evaluated. All data processing was performed in MATLAB R2019b (Mathworks, Inc, Natick, MA, USA).

### Statistical analysis

All data of the RMS and MPF values for each muscle were evaluated in both the H and C groups. The Kolmogorov–Smirnov test was used for normal distribution, and the Mann–Whitney U test was used for comparison. Effect sizes of EMG-RMS, EMG-MPF, MMG-RMS, and MMG-MPF for both the maximal and constant force were calculated using Cohen’s d^40^. Sample sizes and the retrospective two-sided power was evaluated a posteriori (α value of 0.05, two-sided). Maximal tendon reflex was evaluated twice by one observer on the same day for ICC calculation. SEM for each parameter was calculated using the value of ICC^41^. The statistical and power analyses were performed using EZR software (Saitama Medical Center, Jichi Medical University, Japan), which is a graphical user interface for R version 3.5.2 (R Foundation for Statistical Computing, Vienna, Austria)^32^ and ICC analysis were performed using the SPSS software, version 26 (IBM Statistics, Chicago, USA).

## References

[1] Dick JP (2003). The deep tendon and the abdominal reflexes. J. Neurol. Neurosurg. Psychiatry.

[2] Dafkin C, Green A, Kerr S, Veliotes D, McKinon W (2013). The accuracy of subjective clinical assessments of the patellar reflex. Muscle Nerve.

[3] Marshall GL, Little JW (2002). Deep tendon reflexes: a study of quantitative methods. J. Spinal Cord Med..

[4] Lebiedowska MK, Sikdar S, Eranki A, Garmirian L (2011). Knee joint angular velocities and accelerations during the patellar tendon jerk. J. Neurosci. Methods.

[5] Harrop JS (2010). Cervical myelopathy: a clinical and radiographic evaluation and correlation to cervical spondylotic myelopathy. Spine (Phila Pa 1976).

[6] Shimada Y (2006). Spondylolisthesis of the thoracic spine. Case report. J. Neurosurg. Spine.

[7] Mamizuka N, Sakane M, Kaneoka K, Hori N, Ochiai N (2007). Kinematic quantitation of the patellar tendon reflex using a tri-axial accelerometer. J. Biomech..

[8] Hallett M (1993). NINDS myotatic reflex scale. Neurology.

[9] Litvan I (1996). Reliability of the NINDS myotatic reflex scale. Neurology.

[10] Manschot S, van Passel L, Buskens E, Algra A, van Gijn J (1998). Mayo and NINDS scales for assessment of tendon reflexes: between observer agreement and implications for communication. J. Neurol. Neurosurg. Psychiatry.

[11] Frijns CJ, Laman DM, van Duijn MA, van Duijn H (1997). Normal values of patellar and ankle tendon reflex latencies. Clin. Neurol. Neurosurg..

[12] Stam J, van Crevel H (1989). Measurement of tendon reflexes by surface electromyography in normal subjects. J. Neurol..

[13] Stam J, van Crevel H (1990). Reliability of the clinical and electromyographic examination of tendon reflexes. J. Neurol..

[14] Lebiedowska MK, Fisk JR (2003). Quantitative evaluation of reflex and voluntary activity in children with spasticity. Arch. Phys. Med. Rehabil..

[15] Steineman BD, Karra P, Park K (2016). Assessment of patellar tendon reflex responses using second-order system characteristics. Appl. Bionics Biomech..

[16] Tham LK, Abu Osman NA, Wan Abas WA, Lim KS (2013). The validity and reliability of motion analysis in patellar tendon reflex assessment. PLoS ONE.

[17] Stokes MJ, Dalton PA (1991). Acoustic myography for investigating human skeletal muscle fatigue. J. Appl. Physiol..

[18] Keller JL (2019). Self-regulated force and neuromuscular responses during fatiguing isometric leg extensions anchored to a rating of perceived exertion. Appl. Psychophysiol. Biofeedback.

[19] Lozano-Garcia M (2018). Surface mechanomyography and electromyography provide non-invasive indices of inspiratory muscle force and activation in healthy subjects. Sci. Rep..

[20] Cochrane-Snyman KC (2016). Inter-individual variability in the patterns of responses for electromyography and mechanomyography during cycle ergometry using an RPE-clamp model. Eur. J. Appl. Physiol..

[21] Barry DT, Geiringer SR, Ball RD (1985). Acoustic myography: a noninvasive monitor of motor unit fatigue. Muscle Nerve.

[22] Xie HB, Zheng YP, Guo JY (2009). Classification of the mechanomyogram signal using a wavelet packet transform and singular value decomposition for multifunction prosthesis control. Physiol. Meas..

[23] Akataki K, Mita K, Itoh Y (1999). Repeatability study of mechanomyography in submaximal isometric contractions using coefficient of variation and intraclass correlation coefficient. Electromyogr. Clin. Neurophysiol..

[24] Ioi H, Kawakatsu M, Nakata S, Nakasima A, Counts AL (2008). Mechanomyogram and electromyogram analyses during isometric contraction in human masseter muscle. Aust. Orthod. J..

[25] Than C, Seidl L, Tosovic D, Brown JM (2018). Test-retest reliability of laser displacement mechanomyography in paraspinal muscles while in lumbar extension or flexion. J. Electromyogr. Kinesiol..

[26] Fukuhara S, Oka H (2019). A simplified analysis of real-time monitoring of muscle contraction during dynamic exercise using an MMG/EMG hybrid transducer system. Adv. Biomed. Eng..

[27] Walker HK (1990). Clinical Methods: The History, Physical, and Laboratory Examinations.

[28] Kuruoglu R, Oh SJ (1993). Quantitation of tendon reflexes in normal volunteers. Electromyogr. Clin. Neurophysiol..

[29] Xu D, Guo X, Yang CY, Zhang LQ (2015). Assessment of hyperactive reflexes in patients with spinal cord injury. Biomed. Res. Int..

[30] Salazar-Munoz Y (2019). Classification and assessment of the patellar reflex response through biomechanical measures. J. Healthc. Eng..

[31] O'Sullivan R, Kiernan D, Walsh M, O'Brien T, Elhassan Y (2016). Characterisation of the patellar tendon reflex in cerebral palsy children using motion analysis. Ir. J. Med. Sci..

[32] Orizio C (1993). Muscle sound: bases for the introduction of a mechanomyographic signal in muscle studies. Crit. Rev. Biomed. Eng..

[33] Woodward RB, Stokes MJ, Shefelbine SJ, Vaidyanathan R (2019). Segmenting mechanomyography measures of muscle activity phases using inertial data. Sci. Rep..

[34] Marchetti M, Felici F, Bernardi M, Minasi P, Di Filippo L (1992). Can evoked phonomyography be used to recognize fast and slow muscle in man?. Int J Sports Med..

[35] Burke JR, Schutten MC, Koceja DM, Kamen G (1996). Age-dependent effects of muscle vibration and the Jendrassik maneuver on the patellar tendon reflex response. Arch. Phys. Med. Rehabil..

[36] Richardson JK (2002). The clinical identification of peripheral neuropathy among older persons. Arch. Phys. Med. Rehab..

[37] Chandrasekhar A, Abu Osman NA, Tham LK, Lim KS, Wan Abas WA (2013). Influence of age on patellar tendon reflex response. PLoS ONE.

[38] Jaskolska A, Brzenczek W, Kisiel-Sajewicz K, Kawczynski A, Marusiak J, Jaskolski A (2004). The effect of skinfold on frequency of human muscle mechanomyogram. J Electromyogr Kinesiol..

[39] Madeleine P, Vedsted P, Blangsted AK, Sjogaard G, Sogaard K (2006). Effects of electromyographic and mechanomyographic biofeedback on upper trapezius muscle activity during standardized computer work. Ergonomics.

[40] Lee DK (2016). Alternatives to P value: confidence interval and effect size. Korean J. Anesthesiol..

[41] Stratford PW, Goldsmith CH (1997). Use of the standard error as a reliability index of interest: an applied example using elbow flexor strength data. Phys Ther..

